# Pulmonary renal syndrome in a child with coexistence of anti-neutrophil cytoplasmic antibodies and anti-glomerular basement membrane disease: case report and literature review

**DOI:** 10.1186/1471-2369-14-66

**Published:** 2013-03-22

**Authors:** Radovan Bogdanović, Predrag Minić, Jasmina Marković-Lipkovski, Nataša Stajić, Nataša Savić, Milan Rodić

**Affiliations:** 1 Faculty of Medicine, University of Belgrade, Belgrade, Serbia; 2 Institute of Mother and Child Healthcare of Serbia “Dr Vukan Čupić”, 8 R Dakica Str, Belgrade, 11070, Serbia

**Keywords:** Pulmonary renal syndrome, Children, Anti-myeloperoxidase anti-neutrophil cytoplasmic antibodies, Anti-glomerular basement membrane disease

## Abstract

**Background:**

Pulmonary renal syndrome (PRS), denoting the presence of diffuse alveolar hemorrhage and glomerulonephritis as manifestations of systemic autoimmune disease, is very rare in childhood. The coexistence of circulating anti-neutrophil cytoplasmic antibody (ANCA) and anti-glomerular basement membrane (GBM) disease in children affected by this syndrome is exceptional, with unfavorable outcome in five out of seven patients reported to date. We describe a child with PRS associated with both circulating anti-myeloperoxidase (anti-MPO) ANCA and anti-GBM disease on renal biopsy who was successfully treated with immunosuppressive therapy.

**Case presentation:**

A 10-year old girl presented with fever, fatigue, malaise, and pallor followed by hemoptysis and severe anemia. Diffuse alveolar hemorrhage was revealed on fiberoptic bronchoscopy. Renal findings consisted of microscopic hematuria, moderate proteinuria, and anti-GBM disease on renal biopsy. ANCA with anti-MPO specificity were present whereas anti-GBM antibodies were on borderline for positivity. Methyl-prednisolone pulses followed by prednisone led to cessation of hemoptysis, marked improvement of lung fuction, and normal finding on chest x-ray within 10 days. An immunosuppressive regimen was then given consisting of prednisone daily for 4 weeks with subsequent taper on alternate day, i.v. cyclophosphamide pulses monthly for 6 doses, followed by mycophenolate mofetil that resulted in normal lung function tests, hemoglobin concentration, and anti-MPO level within four subsequent weeks. During 10-months of follow-up she remained well, her blood pressure and renal function tests were normal, and proteinuria and hematuria gradually resolved.

**Conclusion:**

We report a child with an exceptionally rare coexistence of circulating ANCA and anti-GBM disease manifesting as PRS in whom renal disease was not the prominent part of clinical presentation, contrary to other reported pediatric patients. A review of literature on disease with double positive antibodies is also presented. Evaluation of a patient with PRS should include testing for presence of different antibodies. An early diagnosis and rapid institution of aggressive immunosuppressive therapy can induce remission and preserve renal function. Renal prognosis depends on the extent of kidney injury at diagnosis and appropriate treatment.

## Background

The term pulmonary renal syndrome (PRS) describes the presence of diffuse alveolar hemorrhage (DAH) and glomerulonephritis as manifestations of multisystemic autoimmune disease often resulting in severe, life-threatening condition requiring urgent, aggressive treatment [1,2]. It is very rare in children. The most common reported causes are systemic lupus erythematosus, anti-neutrophil cytoplasmic antibody (ANCA)-associated vasculitis (AAV), anti-glomerular basement membrane (GBM) disease, and Henoch-Schonlein purpura [3]. Circulating antibodies against GBM and ANCAs are both associated with crescentic glomerulonephritis (CGN) and DAH [4,5]. Their coexistence (double or dual positivity) was found in a subset of adult patients at disease presentation indicating a pathogenic link [6-13]. In children, this coexistence is extremely rare with only seven patients reported to date, five of them had an unfavorable outcome [6,14-18].

We present an exceptional case of a 10-year old girl with DAH and focal necrotizing CGN associated with circulating anti-myeloperoxidase (anti-MPO) ANCA and concomitant anti-GBM disease on renal biopsy who was successfully treated with immunosuppressive therapy. Contrary to other reported pediatric patients, renal disease was not the prominent part of clinical presentation. Pathogenesis, clinical course, treatment and outcome of disease with double positivity are discussed based on a review of reports in adults and children.

## Case presentation

**Figure 1:**
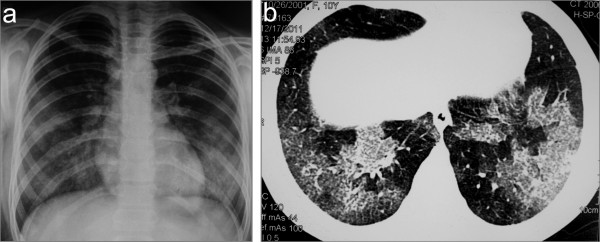
Chest X-ray (a) and CT scan (b) showing bilateral lung infiltrates as a result of diffuse alveolar hemorrhage.

Previously healthy 10-year old girl presented with intermittent fever up to 38,5°C, fatigue, malaise, occasional headaches, legs pain and pallor. The symptoms did not prompt her or her parents to seek medical help. Two weeks later she began to cough, when laboratory investigation showed severe anemia with hemoglobin of 44 g/l and she was admitted to the local hospital. Chest X-ray, echocardiography and ultrasound examination of abdomen were normal. After receiving transfusion of packed red blood cells (RBC) she was referred to regional hospital. During 2 weeks stay she received packed RBC transfusion on two occasions but moderate anemia (hemoglobin up to 94 g/l) persisted. There were no signs of hemolysis and bone marrow examination was normal. Urinalysis showed microscopic hematuria and persistent mild to moderate proteinuria (+ to ++ on dipstick) with normal renal function tests (urea 4.5 mmol/l, creatinine 47 μmol/l). A week after admission she started with dry cough again and began to expectorate blood-tinged sputum. On physical examination, inspiratory crackles over both lower lung fields were noted. Her anemia has worsened (hemoglobin 72 g/l), chest X-ray showed bilateral patchy pulmonary infiltrates (Figure 1a) and active pulmonary hemorrhage was suspected. This was supported by finding of patchy areas of ground-glass opacities on chest computed tomography scan (Figure 1b) suggesting diffuse alveolar hemorrhage. Therapy with prednisone, 20 mg/day was started and she was transferred to our hospital.

On admission, she was pale with body height (149 cm) and body weight (40 kg) at 90. Percentile. Inspiratory crackles were present bilaterally over her lower lung fields, but the rest of physical examination was unremarkable. Blood pressure (BP) was 90/60 mmHg. Chest X-ray showed bilateral patchy pulmonary infiltrates in the middle and lower lung fields. Flexible fiber optic bronchoscopy revealed the presence of bilateral alveolar bleeding with progressively more hemorrhagic samples obtained by consecutive aliquots during bronchoalveolar lavage. The cytological analysis of these specimens showed presence of numerous hemosiderin-laden macrophages. Pulmonary function testing showed restrictive lung disease pattern (vital capacity – VC 71% and total lung capacity – TLC 74% predicted). Blood counts were: Hb 104 g/l, RBC 3.8×10^12^/l, WBC 11.1×10^9^/l, Plt 411×10^9^/l, Rtc 3.12%. Serum urea and creatinin levels were normal. Urinalysis showed microscopic hematuria (8–10 RBC/hpf) and mild proteinuria (474 mg/24 h). Presence of pulmonary and renal manifestations suggested further serological testing. Antinuclear antibodies (ANA) and anti-double stranded DNA (anti-dsDNA) antibodies were negative, and C3 and C4 components of complement were normal. Indirect immunofluorescence (IIF) revealed perinuclear ANCA (p-ANCA) at a titer of 1:80 with antimyeloperoxidase (anti-MPO) ANCA specifity by ELISA (52.7 U/ml, normal <5 U/ml); IIF for cytoplasmic ANCA (c-ANCA), and ELISA for antiproteinase 3 (anti-PR3)-ANCA were negative, but anti-glomerular basement membrane (anti-GBM) antibodies titer (ELISA) was on borderline for positivity (19.7 RU/ml, normal <20 RU/ml). She was immediately given i.v. methylprednisolone (MP) pulses (600 mg/m^2^/day) for 3 days, followed by prednisolone, 40 mg/day, which resulted in cessation of hemoptysis, marked improvement of lung function (VC 90%, TLC 91%) and normal finding on chest X-ray within 10 days.

**Figure 2:**
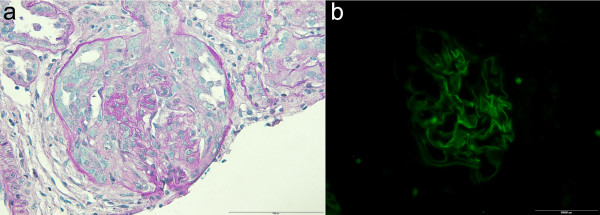
**Light microscopy and immunofluorescent findings on renal biopsy. (a)** Fibrinoid necrosis of GBM, cellular crescentic formation, and slight periglomerular limpho-monocytic infiltration (PAS, ×400); **(b)** Immunofluorescence: fine linear pattern along GBM stained with anti IgG antibody (×200).

However, moderate anemia persisted together with moderate proteinuria (638 mg/24 h) and microscopic hematuria. Percutaneous renal biopsy was perfomed and tissue samples were analyzed by light microscopy (LM) and immunofluorescence (IF). LM showed cellular crescents in 4 out of 25 glomeruli and fibrinoid necrosis in 1 of 25 glomeruli (Figure 2a), with lymphomonocytic interstitial infiltration around glomeruli with crescents and perivascular infiltration of small arterioles; IF showed diffuse global linear staining of the GBM for IgG (Figure 2b) accompanied by segmental linear to granular staining for C3, suggesting anti-GBM disease [5,19,20]. Diagnosis of pulmonary-renal syndrome caused by anti-GBM disease and coexistent AAV was made. The treatment was then changed to a regimen consisting of prednisone (60 mg/day) for 4 weeks with subsequent taper to 30 mg on alternate day; i.v. cyclophosphamide (CY) pulses (0.5–0.75 g/m^2^) were given monthly for total of 6 doses, followed by mycophenolate mofetil (MMF; 600 mg/m^2^ b.d.) [19,21]. Four weeks after starting this treatment her hemoglobin level (132 g/L) and pulmonary function tests (FVC 100%, FEV1 97%) were normal, p-ANCA/anti-MPO ANCA returned to normal and anti-GBM antibodies were undetectable. During the 10-month follow-up she remained well, her BP and renal function test remained normal. Proteinuria remained unchanged during the first 8 months but gradually resolved thereafter, whereas two episodes of macroscopic hematuria on two occasions were occured, during unspecific febrile illnesses. At the latest follow-up visit, 10 months after starting treatment, her body weight was 44 kg, BP was 96/58 mmHg, with normal findings on physical examination and normal pulmonary function tests. Her complete blood count was normal, whereas 4–6 RBC/hpf in urine sediment were only present. Blood and urine laboratory values were as follows: urea 4.5 mmol/l, creatinine 59 μmol/l, p-ANCA negative; anti-MPO ANCA 3.5 U/ml; anti-GBM antibodies undetectable; proteinuria 116 mg/24 h. Her drug therapy consisted of prednisone 15 mg on alternate day, and MMF dose was reduced to 750 mg/day.

## Discussion

The combination of DAH and acute or rapidly progressive (crescentic) glomerulonephritis, known as Goodpasture syndrome, is used interchangeably with PRS since several different pathogenic mechanisms underlie this clinical entity [16,22]. PRS most commonly results from AAV or anti-GBM disease but is also described in lupus, Henoch-Schönlein purpura, rheumathoid arthritis and other connective tissue diseases [2].

In the anti-GBM disease the deposition of autoantibodies in the glomerular and alveolar basement membranes induces glomerulonephritis and pulmonary hemorrhage. The target antigen for the autoantibodies is the non-collagenous-1 (α3NC1) domain of type IV collagen in the GBM, with changes in structure of the α345 hexamer leading to conformation change that elicits this autoimmune response [23]. In renal biopsies, linear positivity for IgG along the GBM indicates the direct pathogenetic relevance of this antibody [5].

ANCA-associated systemic vasculitides are characterized by necrotizing inflammation of the small vessels in conjunction with ANCA directed to myeloperoxidase or proteinase-3 [24]. It is shown that ANCA IgG can activate neutrophils and monocytes through Fc receptor and Fab2 binding leading to their adhesion to endothelial cells and release of cytotoxic factors which results in inflammatory injury of endothelial cells and vessel walls [5,25].

Several studies in adults reported patients with concurrence of ANCA and anti-GBM antibodies. Approximately 5–14% of ANCA positive patients also had positive anti-GBM antibodies [6,10,26], and 21–38% of anti-GBM antibody-positive patients also had positive ANCAs [6-10,26,27], with specificity to MPO in at least two thirds of them [6-11,26,27]. The relatively high incidence of such dual positivity indicates a pathogenic link, which still has to be unraveled. Several pathogenetic hypothesis have been proposed to explain this coexistence, the most attractive one suggests an ANCA development as the initial event, with ANCA-associated mechanisms leading to the exposure of the otherwise hidden α-3(IV)NC1 antigen and triggering anti-GBM antibody production [11,23,28]. This hypothesis is supported by the reports on sequential positivity of ANCA followed by anti-GBM antibodies [26,29], by the strong association between elevated ANCA titers and anti-GBM disease [14,19], and by the recent findings that anti-PR3 and/or anti-MPO antibodies were always detected long before anti-GBM antibodies in anti-GBM disease patients [30]. However, the opposite sequence in double positive patients is also described [31-33].

There are reports that dual positivity in anti-GBM disease may be related to a different clinical course, at least in some patients. Older studies showed more favorable course with significant renal recovery after initiating dialysis [6,7,34], but more recent reports concluded that renal prognosis is comparable to anti-GBM disease and is worse compared to patients with anti-MPO antibodies [10,11]. It has also been noted that incidence of pulmonary hemorrhage is lower in adult double-positive patients than in patients with ANCA-negative anti-GBM disease [6], and that dual positivity is associated with lower anti-GBM titers [6,7,27]. While pure anti-GBM disease is generally considered monophasic, non-relapsing illness [5,10,12,19], AAV has a recurrence rate of 30–60%, demanding maintenance therapy after induction of remission [35]. Dual positive patients have higher relapse rate then those with anti-GBM antibodies alone [6,9]. This suggests that an early indicator of disease relapse is needed in monitoring disease activity in double-positive patients, such as anti-hLAMP2 antibody [36,37].

Optimal therapy for double-positive patients has not been identified yet. Because of the risk of serious disease with multiple organ involvement and possible relapsing course, the addition of plasmapheresis to an immunosuppressive regimen given for AAV with continuing immunosuppression with azathioprine after 3–6 months of cyclophosphamide is recommended for these patients [38,39].

In most adult double positive patients the reported outcome was poor both in terms of the recovery of renal function and mortality [2,6,10,11]. The best predictor for renal survival was renal function at diagnosis: recovery of renal function in dialysis dependent patients is exceptionally rare [7,10,40] as well as in those with serum creatinine exceeding 500 μmol/l despite undergoing treatment [6,7,10-12,34]. In patients who were not dialysis dependent at diagnosis, renal function depended on histological severity of disease [34]. Patients with pulmonary hemorrhage had significantly higher mortality compared to those without pulmonary hemorrhage [10]. The recurrence of disease activity with elevation of MPO-ANCA can occur many months after clinical remission, affecting either both lungs and kidneys or only one of these organs [6,12,13,31,32,41]. Most relapses responded well to methylprednisolone and/or cyclophosphamide therapy [12,36].

The initial symptoms in our patient were similar to those often preceding AAV or may represent a possible infectious trigger either for one or both diseases [19,20]. This was followed by pulmonary hemorrhage with accompanying severe anemia. However, renal disease was relatively mild consisting of microscopic hematuria and mild to moderate proteinuria. Renal biopsy showed focal CGN with only occasional extraglomerular vasculitic changes, but without chronic glomerular or tubulointerstitial lesions. Fibrinoid necrosis with extraglomerular vasculitic changes, associated with positive pANCA/anti-MPO antibodies indicated pauci-immune ANCA disease whereas intense linear IgG staining of the GBM pointed out to an anti–GBM disease [5,19,20], irrespective of normal (though high-normal) serum anti-GBM antibodies level [42]. Only active lesions were present implying the recent onset of disease. Good clinical response of pulmonary disease to corticosteroid treatment and clinically and pathologically mild to moderate renal disease led us to withhold plasmapheresis from recommended treatment regimen [19,21]. The subsequent course was very favorable in view of pulmonary symptoms, global renal function, rapid disappearance of anti-GBM antibodies and normalization of anti-MPO antibodies. However, the substantially unchanged proteinuria and bouts of macroscopic hematuria, that could reflect continuing vasculitic disease activity despite normal anti-MPO antibody titer [43], led us to introduce mycophenolate mofetil after stopping cyclophosphamide therapy [19], as an adjunct to the above regimen. One must assume that both the stability of pulmonary remission and lately achieved complete remission were related to an early intensive corticosteroid and CY therapy, followed by MMF.

The coexistance of anti-GBM antibodies and ANCAs is extremely rare in children. Searching PubMed, we were able to retrieve only seven pediatric cases reported to date, either within case series [6,18] or as single case reports [14-17]. Their main features together with those in our patient are presented in Table 1. Six out of eight patients were females, aged between 4–17 years. PRS was presenting feature in all except in the youngest patient [17]. Preceding constitutional symptoms or different organ involvement were present in five of them. Crescentic glomerulonephritis with positive IgG linear straining along the GBM was found in all eight patients, with percentage of crescents ranging from 13% to 90% in kidney biopsy samples. In one patient, an 8-year old girl [18], circulating anti-GBM antibodies were not detected whereas they were at the upper limit of normal in our patient, despite prominent linear IgG staining in the GBM in both of them. It has been estimated that 2–3% of patients with anti-GBM disease may have circulating antibody levels that are undetectable by standard assays, but can be detected by a more sensitive method [42]. In these cases, diagnosis of anti-GBM disease may be based on intense linear staining of the GBM after exclusion of other conditions, also presenting with linear staining, but extremely rare in children [5,42]. Only one patient had c-ANCA with anti-PR-3 specifity, whereas p-ANCA/anti-MPO antibodies were found in other seven patients. Although MPO specifity is the predominant pattern found in dual positivity, this phenomenon can be seen either with MPO or PR3 [6,32]. All survived patients were treated by combination of immunosuppressive drugs and all except our patient received plasmapheresis.

**Table 1:** Main features of the reported double-positive pediatric patients

**Patient [ref]**	**Age (yr, sex)**	**Initial symptoms**	**Hb, g/dl**	**S-Cr, mg/dl**		**Anti-GBM Abs**	**ANCA type**	**Crescents, %**	**Treatment**	**F/U**	**Outcome**
				**onset F/U**							
1 [14]	17, f	URTI	~ 6.0	12.5	-	+	p/MPO	90	supportive	-	Death at presentation
2 [15]	12, f	-	7.4	0.5	0.3	+	p/MPO	13	MP, PE, CY	few weeks	Normal GFR
3 [16]	12, m	fever,rash, arthritis	10.6	7.4	2.1	+	p/MPO	>60	PE, MP, CY Pdn, MMF	18 mos	GFR 58.5 ml/min
4 [17]	4, f	epistaxis	n.a.	2.7	0.8	+	p/MPO	62	PE, MP, Pdn, CY	9 mos	Normal GFR
5 [18]	8, f	sore throat	2.8	7.7	-	-	p/MPO	83	supportive	-	Death at presentation
6 [6]	13, m	rash, sinusitis	n.a.	D	D	+	c/PR3	+	PE, Pdn, CY	n.a.	ESRD
7 [6]	17, f		n.a.	D	D	+	p/MPO	20	PE, CY, Pdn, Aza	n.a.	ESRD
8 [this report]	10, f	fever, malaise, legs pain	4.4	0.53	0.66	±	p/MPO	16	MP, CY, Pdn, MMF	10 mos	Normal GFR

*URTI*, upper respiratory tract infection; *F/U*, follow-up; *Abs*, antibodies; *p*, perinuclear; *MPO*, myeloperoxidase; *PR3*, proteinase-3; *MP*, methyl-prednisolone; *PE*, plasma exchange; *CY*, cyclophosphamide; *GFR*, glomerular filtration rate; *Pdn*, prednisone; *MMF*, mycophenolate mofetil; *Aza*, azathioprine; *ESRD*, end-stage renal disease; *D*, dialysis; *S-Cr*, serum creatinine concentration; *GBM*, glomerular basement membrane;

*ANCA*, anti-neutrophil cytoplasmic antibodies; *n.a*., not available; +, positive/present; -, negative/absent.

In both AAV and anti-GBM disease the addition of plasmapheresis to initial immunosuppressive therapy is indicated in patients with severe renal dysfunction or with DAH [19,20,38,39]. The recommendation for plasmapheresis in double positive patients is based on the rationale for the treatment of anti-GBM disease [38,39]. The observation of worse outcome in patients with double positivity compared to those with either AAV or anti-GBM disease alone [11] may add further support to this strategy. However, it is unknown whether patients with milder alveolar hemorrhage require plasmapheresis [38]. According to these criteria, plasmapheresis was indicated in our patient. However, quick favorable response to intensive immunomodulating therapy as well as the absence of severe renal dysfunction, led us to withhold plasmapheresis from the standard treatment approach. In a few anti-GBM disease patients having DAH and normal renal function, good outcome was seen without adding plasmapheresis to immunosuppression [44]. However, we could not find a similar case among AAV patients or double positive patients reported to date. The unfavorable outcome in children with double positivity and DAH, having either low or high percentage of glomeruli with crescents [6,14,18], strongly remind of the serious nature of the disease for which we do not still have robust indicator to assist with optimal personalized therapy.

The outcome of reported pediatric cases is not different from that in adults: two patients died at presentation from massive pulmonary hemorrhage [14,18]; two patients, both dialysis dependent at presentation, remained in end-stage renal disease (ESRD) [6]; one patient, whose treatment was delayed 5 months after clinical onset, improved renal function [16]; three patients, including ours, had normal renal function several weeks to 10 months after onset of disease [15,17]. Our patient and a 12-year old girl reported by Paueksakon et al. [15] had normal renal function at onset with cellular crescents in only 16% or 13% of glomeruli, respectively. These two patients may resemble to a small subgroup of younger adult patients with anti-GBM disease presenting with milder kidney involvement and an excellent prognosis despite severe pulmonary hemorrhage [44]. The third patient, 4-year-old girl, who is the youngest patient, had advanced disease, as shown by 62% of glomeruli with crescents and 33% globally sclerosed, responded well to treatment [17], similarly to very rare adult patients [7,10,40]. On the other hand, a 17-year old girl with only 20% of crescents on biopsy was dialysis dependent at presentation and remained in ESRD despite plasmapheresis and aggressive immunosuppression. No other details are given except that she had significant glomerular sclerosis [6]. One patient had relapse with pulmonary hemorrhage six months after onset [6].

## Conclusion

The pulmonary renal syndrome is very rare in children but represents a serious medical emergency with significant morbidity and mortality. An early diagnosis is crucial and laboratory investigation should be directed toward testing for presence of different antibodies. The coexistence of anti-GBM antibodies and ANCAs is exceptionally rare in children. Rapid institution of plasmapheresis and aggressive immunosuppressive therapy can induce remission and preserve renal function in dual positive patients with PRS. Prolonged therapy, long-term follow-up and close monitoring are necessary as relapses may occur. Renal prognosis depends on the extent of kidney injury at diagnosis and appropriate treatment.

## Consent

Written informed consent was obtained from the child’s father for publication of case report and any accompanying images.

## Abbreviations

AAV: ANCA-associated vasculitis; ANA: Antinuclear antibodies; ANCA: Anti-neutrophil cytoplasmic antibodies; BP: Blood pressure; CGN: Crescentic glomerulonephritis; CT: Computed tomography; CY: Cyclophosphamide; DAH: Diffuse alveolar hemorrhage; ds-DNA: Double stranded deoxyribonucleic acid; ELISA: Enzyme linked immunoassay; ESRD: End stage renal disease; GBM: Glomerular basement membrane; IF: Immunofluorescence; IIF: Indirect immunofluorescence; LM: Light microscopy; LM: Light microscopy; MMF: Mycophenolate mofetil; MPO: Myeloperoxidase; PR3: Proteinase-3; PRS: Pulmonary renal syndrome; RBC: Red blood cells; TLC: Total lung capacity; VC: Vital capacity.

## Competing interests

The authors declare that they have no competing interests.

## Authors’ contributions

RB, PM, NS, NS, and MR were the physicians who treated the patient in this report. JM-L performed the pathology studies. The manuscript was prepared by RB. All authors participated in discussions about the manuscript and approved the final version.

## Pre-publication history

The pre-publication history for this paper can be accessed here:

http://www.biomedcentral.com/1471-2369/14/66/prepub
